# *Plasmodium chabaudi *limits early *Nippostrongylus brasiliensis*-induced pulmonary immune activation and Th2 polarization in co-infected mice

**DOI:** 10.1186/1471-2172-10-60

**Published:** 2009-12-01

**Authors:** Marieke A Hoeve, Katie J Mylonas, Karen J Fairlie-Clarke, Simmi M Mahajan, Judith E Allen, Andrea L Graham

**Affiliations:** 1 Institute of Immunology and Infection Research, School of Biological Sciences, University of Edinburgh, Edinburgh, EH9 3JT, UK; 2 Department of Ecology & Evolutionary Biology, Princeton University, Princeton, New Jersey, 08544, USA

## Abstract

**Background:**

Larvae of several common species of parasitic nematodes obligately migrate through, and often damage, host lungs. The larvae induce strong pulmonary Type 2 immune responses, including T-helper (Th)2 cells as well as alternatively activated macrophages (AAMφ) and associated chitinase and Fizz/resistin family members (ChaFFs), which are thought to promote tissue repair processes. Given the prevalence of systemic or lung-resident Type 1-inducing pathogens in geographical areas in which nematodes are endemic, we wished to investigate the impact of concurrent Type 1 responses on the development of these Type 2 responses to nematode larval migration. We therefore infected BALB/c mice with the nematode *Nippostrongylus brasiliensis*, in the presence or absence of *Plasmodium chabaudi chabaudi *malaria parasites. Co-infected animals received both infections on the same day, and disease was assessed daily before immunological measurements were taken at 3, 5, 7 or 20 days post-infection.

**Results:**

We observed that the nematodes themselves caused transient loss of body mass and red blood cell density, but co-infection then slightly ameliorated the severity of malarial anaemia. We also tracked the development of immune responses in the lung and thoracic lymph node. By the time of onset of the adaptive immune response around 7 days post-infection, malaria co-infection had reduced pulmonary expression of ChaFFs. Assessment of the T cell response demonstrated that the Th2 response to the nematode was also significantly impaired by malaria co-infection.

**Conclusion:**

*P. c. chabaudi *co-infection altered both local and lymph node Type 2 immune activation due to migration of *N. brasiliensis *larvae. Given recent work from other laboratories showing that *N. brasiliensis*-induced ChaFFs correlate to the extent of long-term lung damage, our results raise the possibility that co-infection with malaria might alter pulmonary repair processes following nematode migration. Further experimentation in the co-infection model developed here will reveal the longer-term consequences of the presence of both malaria and helminths in the lung.

## Background

Many prevalent species of parasitic nematodes - such as *Ascaris lumbricoides*, which infects over a billion people [1], or *Necator americanus*, the most geographically widespread of the human hookworms [2] - migrate through host lungs as larvae. Lung tissue is ruptured as the larvae burst out of the blood vessels to enter the alveolar spaces. Although this process is typically asymptomatic in humans, it can also be associated with acute respiratory distress or longer term complications [3]. For example, infection with lung-migrating helminths has been associated with bronchial hyper-reactivity and other asthma symptoms among children in China [4] and Brazil [5].

The rodent parasite *Nippostrongylus brasiliensis *(*Nb*) has proven a valuable laboratory model for nematode migration through the host body. In mice, L3 larvae injected into the skin migrate via the lungs to the small intestine, where the parasites develop into adults [6]. Peak abundance of *Nb *larvae in the lung occurs around 2 days post-infection (pi) in many strains of mice [7]. The lung migratory stage of *Nb *is associated with a strong local Type 2 inflammatory response that includes T-helper (Th)2 cells, eosinophils and basophils [8,9]. Alternatively-activated macrophages (AAMφ) have also been identified as a major component of the pulmonary response to *Nb *infection [10,11]. AAMφ are characterised by IL-4/IL-13-dependent production of chitinase and Fizz/resistin family members (ChaFFs) including RELMα (also known as Fizz1), the chitinase-like protein Ym1, and Arginase-1 [12-15], and all three proteins are consistently observed in the *Nb *infected lung [10,11,16-18]. Arginase-1 is the counter-regulatory enzyme to iNOS and can thus act to suppress NO production and Type 1 effector function. Arginase-1 also has well documented roles in tissue repair [19,20] and has recently been implicated as an anti-nematode effector molecule [21]. The functions of RELMα and Ym1 are less well understood but, like Arginase-1, they have been strongly implicated in the response to injury [22-24] and have putative roles in the repair process, including extra-cellular matrix deposition and angiogenesis [25,26]. However, recent data have shown that RELMα and macrophage-derived arginase can also negatively regulate Th2 effector responses and thus limit the pathology associated with overzealous repair [27-29].

Although not formally proven, the association of Arginase-1, RELMα, and Ym1 with the tissue repair process suggests that in the context of nematode infection, ChaFFs, potentially produced by AAMφ, may be required to orchestrate the repair of damage caused by larval migration in order to restore lung integrity. Two recent papers have highlighted the potential for *Nb *migration to damage the lung with potentially long term consequences [16,18]. Both studies document haemorrhaging of lung tissue and sustained increases in airway hyper-responsiveness. A striking novel observation in these studies is that *Nb *causes disruption of the alveolar architecture that is consistent with pulmonary emphysema many weeks after infection. Dysregulated, AAMφ-mediated repair of the damage caused by the nematodes may be responsible for such detrimental outcomes [16].

Helminths with lung migratory stages are often co-endemic with Type 1-inducing parasites such as malaria [30-32]. Given the potential for cross-regulation between Type 1 and Type 2 immune responses, we wished to use mouse models to investigate the consequences of co-infection for the pulmonary Type 2 immune responses induced by nematode migration. We chose to focus on *Nb *and a rodent malaria, *Plasmodium chabaudi chabaudi *(*Pcc*), that induces a potent Type 1 immune response and non-lethal infection [33]. We challenged hosts simultaneously with these two acute infections, thus demanding polarized, conflicting immune responses at the same point in time. In addition, we expected *Nb-Pcc *co-infection to induce conflicting responses in the same anatomical location, because malaria-infected red blood cells (RBCs) of many species, including *Pcc*, adhere to endothelial cells of the microvasculature of the lung [34-36]. Furthermore, malaria itself has been shown to cause lung injury [37,38]. Thus, we expected the lung and draining (thoracic) lymph nodes to be potential sites of strong interactions between *Nb *and *Pcc*. The idea that helminth-malaria co-infection may impose Type 1-Type 2 immunological conflict is not new [30], nor is the idea that parasitic co-infection may alter the severity of pulmonary disease [39,40], but our emphasis on the consequences of malaria for pulmonary Type 2 responses has not previously been explored.

Using these model systems, we assessed production of the ChaFFs, RELMα and Ym1 as primary read-outs of the Type 2 effector response in the lung. We also examined thoracic lymph node (TLN) cytokine profiles, parasitology and systemic pathology, to set the co-infected lung in its whole-organism context. By 7 days pi, malaria infection had significantly reduced the expression of ChaFFs in the lungs of co-infected animals relative to those with *Nb *only. This reduction correlated with changes in Th2 cytokines in the TLN, with co-infected mice producing significantly less IL-13, IL-10 and IL-5 than mice infected with *Nb *only. *Pcc *co-infection thus reduced the extent of pulmonary Type 2 activation and Th2 polarisation in response to *Nb*. Future long-term experiments (up to a year in duration [16]) in the co-infection model established here will explore how helminth migration may interact with malaria infection to affect chronic lung pathology.

## Results

### Nb infection caused loss of both body mass and RBC density but ameliorated Pcc infection

To investigate pulmonary immune polarization during acute helminth-malaria co-infection, on day 0 we infected female BALB/c mice with 200 *Nb *L3 larvae, in the presence or absence of co-infection with 10^5 ^*Pcc-*infected RBCs. Our first goal in developing this model was to characterise the systemic pathology induced by each infection and co-infection. We thus measured body mass (to the nearest 0.1 g), RBC density (billions/mL), and malaria blood parasitaemia daily. We also assessed the presence of malaria parasites in lung tissue of mice culled at 3 or 7 days pi.

**Figure 1:**
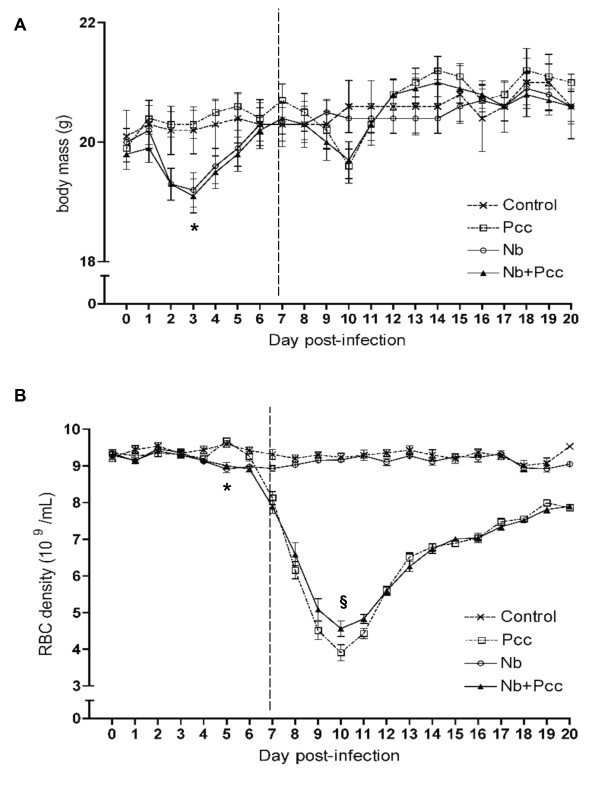
**Pathology, measured as loss of body mass (A) and red blood cell density (B)**. Mice were infected with 200 *Nb *L3 larvae and/or 10^5 ^*Pcc*-infected RBCs on day 0, then weighed daily to the nearest 0.1 g. Daily samples of blood were taken for flow cytometric analysis of RBC density. (A) *Nb *infection induced significant loss of body mass during the first week of infection (lower minimum body mass than mice without *Nb*; *P *< 0.0001). (B) *Nb *also induced significant loss of RBCs that week (lower minimum RBC density to 7 days pi than mice without *Nb*; *P *= 0.0037), whereas *Pcc*-induced RBC loss was mainly apparent during the second week of infection (lower minimum RBC density to 20 days pi than mice without *Pcc; P *< 0.0001). *Nb *co-infection significantly ameliorated this (higher minimum RBC density in co-infected compared to *Pcc*-infected mice; *P *= 0.0003). Means ± SEM from combined results of 3 independent experiments (each with 4-9 mice per group per time point) are shown. The vertical dashed line indicates the two time periods for which minimum weight and RBC density were analysed: up to 7 days pi, and 7-20 days pi, as described in Methods. The symbol * indicates statistical significance of the *Nb *main effect, and § indicates significance of the difference between co-infected and *Pcc *mice.

Consistent with previous reports in rats [41], we found that *Nb *infection induced loss of body mass in mice during the first week of infection: *Nb*-infected mice reached a significantly lower minimum body mass than mice without *Nb *(Fig. 1A; F_1,110 _= 21.1; *P *< 0.0001). This amounted to a mean loss of 0.7 g, or ~3% of body mass, and was observed regardless of malaria co-infection. No further changes in body mass among groups achieved statistical significance, though *Pcc*-infected mice showed an expected dip in weight around day 10 that was unaltered by *Nb*.

In addition to weight loss, *Nb *infection caused RBC densities to be reduced by ~5% (Fig. 1B; effect of *Nb *on minimum RBC density to 7 days pi: F_1,110 _= 8.8; *P *= 0.0037). Unsurprisingly [33], *Pcc *also caused loss of RBCs by 7 days pi (F_1,110 _= 40.2; *P *< 0.0001). Between days 7 and 20 pi, *Nb*-induced RBC loss resolved, but *Pcc *induced further loss of RBCs - up to 60% of original density (Fig. 1B; F_1,110 _= 385.4; *P *< 0.0001). However, this was slightly (~5%) but significantly ameliorated by *Nb *co-infection (Fig. 1B; t_110 _= 4.2; *P *= 0.0003).

**Figure 2:**
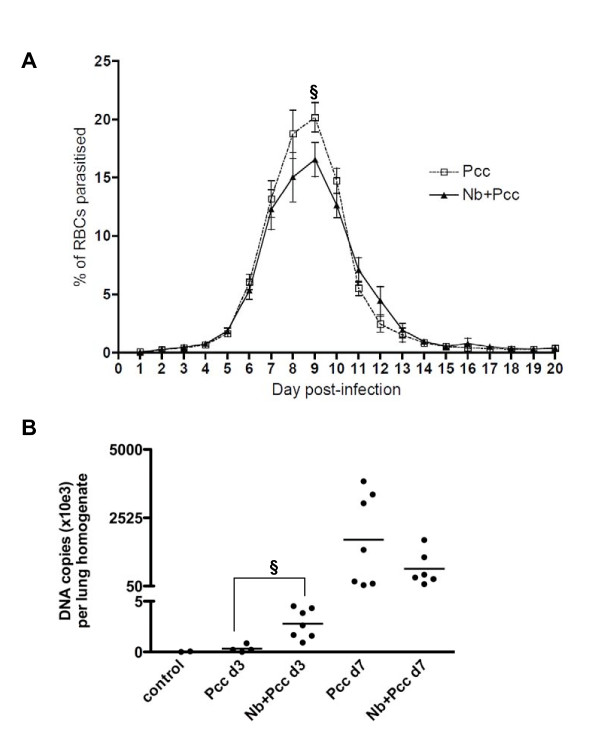
**Malaria parasites in the blood (A) and lungs (B) of *Pcc*-infected and co-infected mice**. (A) Co-infected mice (*Nb+Pcc*) had a lower peak proportion of parasitized RBCs than *Pcc*-infected mice did (*P *= 0.0465), as determined by 1000× microscopic examination of Giemsa-stained thin blood films. Means ± SEM from combined results of 3 independent experiments (each with 4-9 mice per group per time point) are shown. (B) Co-infected mice harboured greater numbers of *Pcc *genome copies per lung sample than *Pcc *mice at 3 days pi (*P *= 0.01), as assessed by real-time PCR of lung homogenates (derived from 75 mg lung) using MSP-1 specific primers. Genome copies were apparent in both *Pcc *infected groups at 7 days pi and the copy numbers no longer differed significantly between groups. This experiment included 4-7 mice per group. The symbol § indicates significance of the difference between co-infected and *Pcc *mice.

Consistent with this slight protective effect of *Nb *on RBC loss during peak *Pcc *infection, *Nb *co-infection was associated with a modest reduction in *Pcc *blood parasitaemia, as determined by microscopic examination of blood films (Fig. 2A; maximum parasitaemia F_1,62 _= 4.13; *P *= 0.0465).

By real-time PCR, the number of *Pcc *genome copies per 75 mg lung homogenate sample was assessed in *Pcc*-infected versus co-infected mice. Malaria parasites were present in the lung of most animals examined at 3 days pi and all animals at 7 days pi (Fig. 2B). At 3 days pi, which is shortly after *Nb *parasites have migrated through the lung [7] and coincident with the loss of body mass in *Nb*-infected mice (Fig. 1A), co-infected mice had more *Pcc *genome copies per lung sample (Fig. 2B; *P *= 0.010). This could be due to enhanced *Pcc *adherence to the lung endothelia [34] during *Nb *co-infection. However, by gross examination we observed substantial haemorrhaging in all *Nb*-infected mice at this time point and thus it is also possible that leakage of blood into the lung tissue increased the number of *Pcc *parasites in day 3 samples. At 7 days pi there was no significant difference between the *Pcc *and the *Nb*+*Pcc *groups.

Gut nematode burden at 3 days pi varied between experiments: for example, *Nb+Pcc *and *Nb *mice bore 56+10 and 35+9 adult nematodes, respectively, in experiment one, versus 6+4 and 1+1 nematodes in experiment two. Such variation has been previously reported [7] and in the present study is likely to be due to the very rapid infection kinetics typically observed for our *Nb *strain, which is not mouse-adapted (e.g., no nematodes remain in the gut at 5 days pi). A difference of a few hours in *Nb *injection times on day 0 and/or in gut sampling times on day 3 could therefore lead to the differences in nematode burden that we observed. Indeed, two lines of evidence suggest that the number of *Nb *larvae moving through the lung was much more consistent than the observed gut burdens. First, there were no significant differences among experiments in the amount of *Nb*-induced weight loss (*P*~0.2) nor RBC loss (*P*~0.4). Furthermore, Type 2 immunological readouts were extremely consistent among experiments. For example, TLN production of IL-4, IL-5, and IL-13 in response to *Nb *infection did not differ significantly among experiments (*P*~0.9, 0.8, and 0.9, respectively). Still, in order to be certain that experimental variations were not confounding any of our conclusions, we controlled for *experiment *in all statistical analyses of combined data (as described in Methods).

### Nb-induced ChaFFs in the lung peaked around 5-7 days pi

Before we undertook studies of Type 2 immune responses in the lung during co-infection, we assessed the time course of *Nb*-induced pulmonary expression of ChaFFs. Previous work has mainly focused on ChaFF mRNA expression in the lungs [10,11,16,17]. We wished to also ascertain protein expression *in situ *after *Nb *infection, to more closely determine the location of these proteins *in vivo*. Female BALB/c mice were infected with 200 *Nb*-L3s, or injected with PBS as a control, and RELMα and Ym1 protein levels were determined in BALF (via Western blots) and lungs (via IHC) at days 3, 5, 7, 15, 20 and 26 pi, to reflect the early events, peak Th2 time point and resolution stages of *Nb *infection [9].

**Figure 3:**
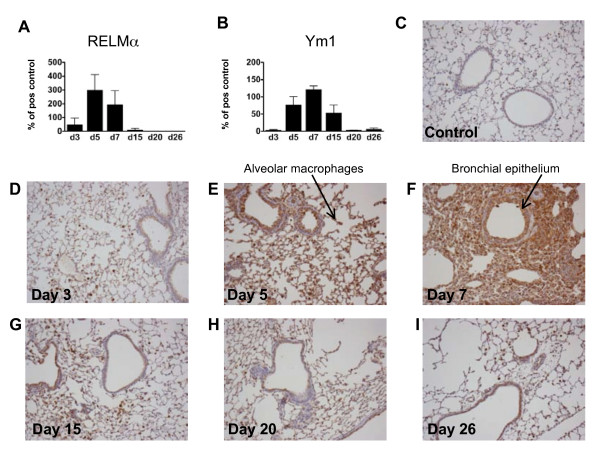
**Time course of protein expression of ChaFFs in *Nb *infected lungs**. Western blot analysis of lung lavages harvested at different time points post *Nb *infection (4 *Nb *mice per timepoint) suggested that the secretion of ChaFFs RELMα (A) and Ym1 (B) peaked around 5-7 days post infection. Data are expressed as percentage of a positive control sample (pooled lung lavages of three *Nb*-infected mice). Representative photomicrographs from *Nb*-infected lung tissue sections stained histologically for Ym1 at the indicated time points, plus an uninfected control lung (C), illustrate peak expression of brown Ym1 staining of epithelial cells and macrophages (E-F).

RELMα and Ym1 were both detected in BALF at 3 days pi, and rose to a peak around 5-7 days pi (Fig. 3A, B). Expression of both proteins dropped off by days 20-26 pi. Histological analysis of Ym1-stained lung sections from infected mice illustrated this peak, with an increase in the intensity and area of anti-Ym1 staining at 5-7 days pi (Fig. 3E, F). These representative micrographs also show the influx of Ym1-positive inflammatory cells into the lung tissue that was evident by day 7 - e.g., alveolar and peribronchial inflammation. Of note, although infiltrating cells were RELMα^+ ^(data not shown) and Ym1^+^, epithelial cells also appeared to be a major source of these molecules. Reece et al. (2006) clearly demonstrate macrophages as sources of these proteins in the lung during *Nb *infection but do not mention epithelial cells [11]. Our data are more consistent with a recent study in which RELMα was localized primarily to epithelial cells in *Nb*-infected mice [29]. Further, several reports on inflamed allergic (asthma model) and fibrotic (bleomycin- or gammaherpes virus-induced) rodent lungs, as well as our own unpublished data, demonstrate expression of both Ym1 and RELMα by epithelial cells [26,42,43]. By 15-26 days pi (Fig. 3G, H, I), Ym1 protein expression had returned to near-background (Fig. 3C), with reduced inflammatory influx and resolution of the thickened and disrupted epithelial layer that was apparent at earlier time points. These data are supportive of the idea that pulmonary activation of AAMφ is a highly dynamic process [44].

### Pcc changed the dynamics of expression of Nb-induced ChaFFs, especially 7 days pi

To assess the effect of *Pcc *co-infection on the dynamics of *Nb*-induced pulmonary AAMφ and Type 2 epithelial cell activation, we next analysed both mRNA and protein expression of two ChaFFs, RELMα and Ym1, at a series of time points during co-infection. We also measured local mRNA expression of iNOS as a marker for Type 1 macrophage activation [19], which might be expected during malaria [45]. Furthermore, we measured mRNA of Type 1 cytokines (IL-12p40, TNF-α, and IFN-γ), as well as mRNA of IL-13, a key cytokine likely to drive production of Type 2 effector molecules such as the ChaFFs [12-15]. We chose to examine the effect of malaria infection around the peak of larvae-induced damage (i.e., ~day 3 pi) [7] and the time of transition to adaptive Type 2 responses (i.e., ~days 5-7 pi) [9,11]. We also assessed a later time point well into the adaptive immune phase: 20 days pi.

**Figure 4:**
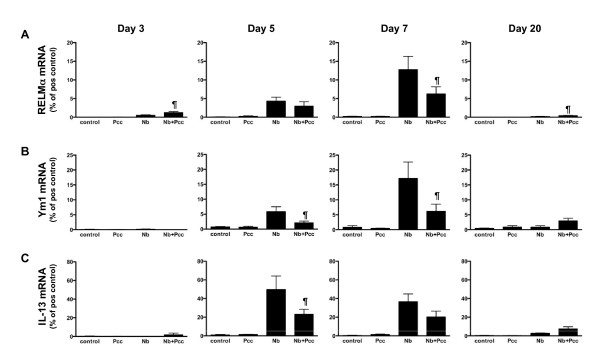
**ChaFF and IL-13 mRNA expression in the lungs of *Nb, Pcc *and co-infected mice**. Real-time RT-PCR of mRNA from lung tissue harvested at 3, 5, 7 or 20 days pi showed that the effect of *Pcc *on *Nb*-induced expression of RELMα (A) and Ym1 (B) changed over time. RELMα was transiently elevated in co-infected (*Nb+Pcc*) compared to *Nb *mice at 3 days pi (*P *= 0.0257), but expression of mRNA for RELMα (A), and Ym1 (B) was then found to be significantly lower in the lungs of *Nb+Pcc *mice than in *Nb *mice by 7 days pi (RELMα, *P *= 0.0113; Ym1, *P *= 0.005). IL-13 mRNA expression (C) largely mirrored this pattern, as it was significantly reduced in *Nb+Pcc *mice compared to *Nb *mice at 5 days pi (*P *= 0.0169). At 20 days pi, however, RELMα (A) was again significantly elevated in *Nb+Pcc *relative to *Nb *mice. (*P *= 0.01) Data are expressed as percentage of positive control samples (for RELMα and Ym1, peritoneal macrophages of a mouse implanted with *Brugia malayi *adult parasites for 3 weeks [72]; for IL-13, spleen of an MHV-68 infected IFNγR-KO mouse [63]). Bars indicate mean ± SEM of 2 combined independent experiments, each with 4-9 mice per group per timepoint. The symbol ¶ indicates significance of the difference between co-infected and *Nb *mice (*P *values included above and in Results text).

Analysis of ChaFF mRNA expression at 3, 5, 7 and 20 days pi suggested that the strongest interactions between *Nb *and *Pcc*-induced responses in lung tissue occurred 5-7 days pi (Fig. 4A, B). Indeed, because days 5-7 pi represented a time of strong ChaFF expression in *Nb *lung (Fig. 3) and day 7 pi coincided with the presence of *Pcc *there (Fig. 2B), it is perhaps unsurprising that days 5-7 pi could be the time of maximum effect of *Pcc *co-infection. At 3 days pi, there was a transient elevation of RELMα mRNA in co-infected compared to *Nb*-infected mice (*P *= 0.0257) that was reversed over the next few days: differences between the groups were not significant at 5 days pi, but RELMα gene expression was significantly reduced in co-infected mice at 7 days pi (Fig. 4A; F_1,25 _= 7.5, *P *= 0.0113, for combined analysis of experiments). Expression of Ym1 mRNA was also significantly lower in co-infected than *Nb *mice at days 5 and 7 pi (Fig. 4B: day 5 *P *= 0.0442; day 7 F_1,25 _= 9.5, *P *= 0.005, for combined analysis of experiments). In agreement with these observations, day 7 expression of Arginase-1 mRNA was significantly lower in the lungs of co-infected mice than in mice that had *Nb *only (data not shown; F_1,24 _= 7.7, *P *= 0.0104). Furthermore, ChaFF expression is known to be driven by engagement of IL-4Rα [24] by IL-4 and/or IL-13; accordingly, IL-13 mRNA expression in the lungs of co-infected mice was significantly reduced compared to *Nb*-infected mice at 5 days pi (Fig. 4C: *P *= 0.0169). This suggests that suppression of IL-13 by *Pcc *may be responsible for the reduced ChaFFs in co-infected mice. At 20 days pi, mRNA expression for RELMα (*P *= 0.0101) was slightly elevated in co-infected relative to *Nb*-only mice, but ChaFF and IL-13 mRNA expression had otherwise largely returned to background levels. Throughout this time course, *Pcc*-infected and uninfected animals expressed little or no mRNA for the ChaFFs.

**Figure 5:**
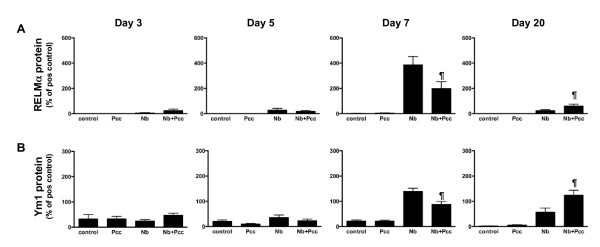
**Western blot analysis of ChaFF protein expression in BALF of *Nb, Pcc *and co-infected mice**. Lung lavages harvested at days 3, 5, 7 and 20 pi indicated that the ChaFF proteins RELMα (A) and Ym1 (B) exhibited temporal dynamics that were similar to those observed for mRNA expression in lung tissue (Fig. 4). In particular, expression of both proteins was significantly lower in the lungs of co-infected (*Nb+Pcc*) than in *Nb *mice at 7 days pi (RELMα *P *= 0.0067 and Ym1 *P *= 0.0104). This pattern had reversed by 20 days pi, with increased ChaFF protein expression in *Nb+Pcc *compared to *Nb *mice (RELMα *P *= 0.0398 and Ym1 *P *= 0.0171). Data are expressed as percentage of a positive control sample (pooled lung lavages of three *Nb*-infected mice). Bars indicate mean ± SEM of combined results of 2 independent experiments, each with 4-9 mice per group per timepoint. The symbol ¶ indicates significance of the difference between co-infected and *Nb *mice (*P *values included above and in Results text).

**Figure 6:**
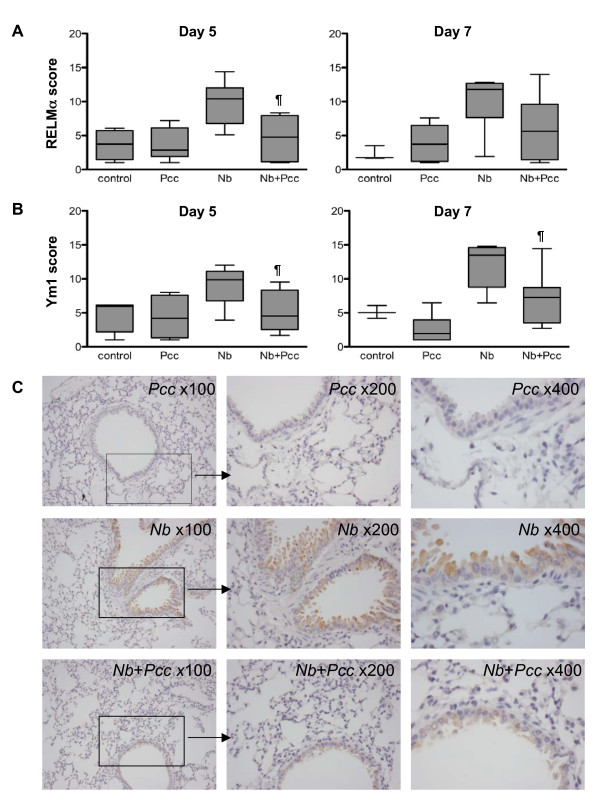
**Immunohistochemical analysis of ChaFF protein expression in the lungs of *Nb, Pcc *and co-infected mice**. Protein expression in lung sections corroborated BALF concentrations of ChaFF proteins observed around 7 days pi, as illustrated by days 5 and 7 pi staining intensity scores for RELMα (A) and Ym1 (B) as well as representative micrographs (at increasing magnification) of day 7 pi slides stained for RELMα (C). Down-regulation of ChaFFs was observed in co-infected mice (*Nb+Pcc*) compared to *Nb *mice; differences were significant for both RELMα and Ym1 at 5 days pi (RELMα *P *= 0.0056 and Ym1 *P *= 0.0153) and for Ym1 at 7 days pi (*P *= 0.0171). Scores were obtained by analyzing bronchial epithelium in ten fields per mouse using a scoring system described in Methods. The whisker-and-box graphs with minimum and maximum values depict results from a representative experiment with 4-9 mice per group. The symbol ¶ indicates significance of the difference between co-infected and *Nb *mice (*P *values included above and in Results text).

In support of the mRNA data, when we analysed protein expression in lung BALF using Western blotting, we saw a significant day 7 pi reduction of both RELMα (Fig. 5A; F_1,25 _= 8.7, *P *= 0.0067, for analysis of combined experiments) and Ym1 (Fig. 5B; F_1,25 _= 7.7, *P *= 0.0104, for analysis of combined experiments) in co-infected mice compared to *Nb*-infected mice. By day 20 pi, the pattern had reversed, with co-infected mice expressing more RELMα (*P *= 0.0398) and Ym1 (*P *= 0.0171) than *Nb *mice. ChaFF expression in *Pcc*-infected mice did not differ from near-null expression in uninfected control mice (Fig. 5A, B). Importantly, BAL cell counts and cellular composition did not differ significantly among groups at any timepoint. There was a non-significant elevation in total numbers of cells in co-infected mice at 3 days pi and in *Nb *mice at 5 days pi, which might partly explain the elevation in ChaFFs at these early timepoints. However, any apparent differences were entirely absent from the 7 and 20 day BAL samples. Therefore, the observed effects on ChaFF protein expression are unlikely to be due to differences in cellular makeup alone, particularly at 7 and 20 days pi. Further, the inflammatory cells may not be the major source of CHaFF proteins in lavage fluid. The pattern and intensity of epithelial cell staining (see Fig 6C) suggests that these cells may be largely responsible for the changes in mRNA and BALF protein.

**Figure 7:**
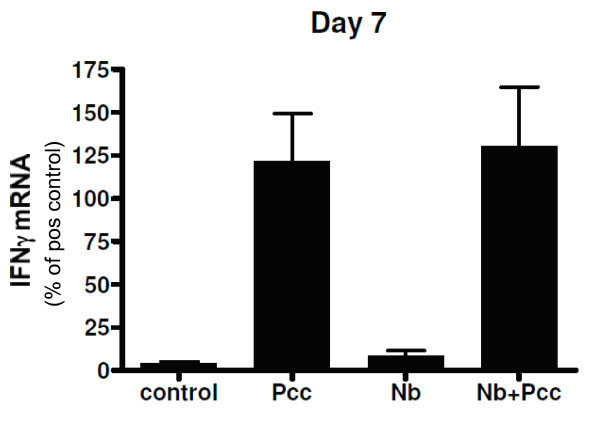
**Lung mRNA for the Type 1 cytokine IFN-γ at 7 days pi**. Real-time RT-PCR of mRNA from lung tissue harvested at 3, 5, 7 or 20 days pi using primers for Type 1 markers iNOS, IL-12p40, TNF-α, and IFN-γ were largely negative. For example, no iNOS nor IL-12p40 was detected in any mice, and although TNF-α was detected at low levels, its expression did not differ among groups of mice. At 7 days pi, however, IFN-γ was significantly upregulated in all mice with *Pcc *infection, with or without *Nb *co-infection, as depicted here. Data are expressed as percentage of a positive control sample (peritoneal macrophages of a thioglycolate-injected mouse [10]). Mean ± SEM from a representative experiment with 4-9 mice per group per timepoint are shown.

IHC scoring of lung sections further confirmed these dynamics. At both day 5 pi and day 7 pi, the intensity of RELMα (Fig 6A) and Ym1 (Fig 6B) staining in the lungs of co-infected animals was reduced compared to *Nb *mice, though the RELMα difference did not achieve statistical significance at day 7 (day 5: RELMα *P *= 0.0056; Ym1 *P *= 0.0153; day 7: RELMα *P *= 0.1011; Ym1 *P *= 0.0171). Representative micrographs from day 7 pi show the reduced intensity of RELMα staining in co-infected animals relative to *Nb *mice and no RELMα staining in *Pcc *mice (Fig. 6C). No differences among groups were detected by IHC at days 3 or 20 pi. Therefore, the expression pattern of ChaFF protein *in situ *was largely in agreement with mRNA in whole lung tissue (Fig. 4) and protein expression in the BALF (Fig. 5). As discussed above for the time course of *Nb *infection (Fig. 3), although Ym1^+ ^and RELMα^+ ^macrophages were present in lung IHC sections, the predominant cell type expressing these molecules appeared to be epithelial cells. Furthermore, lung macrophages in this system do not appear to become classically activated, as assessed by lung mRNA for iNOS and IL-12p40, which were not detectable in any mice at any time point, and TNF-α, which was detectable but at low levels that did not differ among groups (data not shown). However, IFNγ was elevated in the lung tissue of all *Pcc *infected mice, regardless of *Nb *co-infection, at day 7 pi (Fig. 7).

### Cytokine production in local LN largely mirrored pulmonary ChaFF expression

To assess whether pulmonary ChaFF patterns corresponded to immune responses in the draining thoracic lymph node (TLN), we performed *in vitro *culture of TLN cells, in media alone or with antigen or ConA. At 3 and 5 days pi, there were no significant differences in supernatant cytokine concentrations among infection groups. However, by 7 days pi, differences among the groups were apparent, as outlined below. No antigen-specific responses were observed (i.e., no cytokines in excess of spontaneous secretion in media alone) until 20 days pi (discussed below). Spontaneous and ConA-induced cytokines exhibited identical patterns, though spontaneous responses were of lower magnitude.

**Figure 8:**
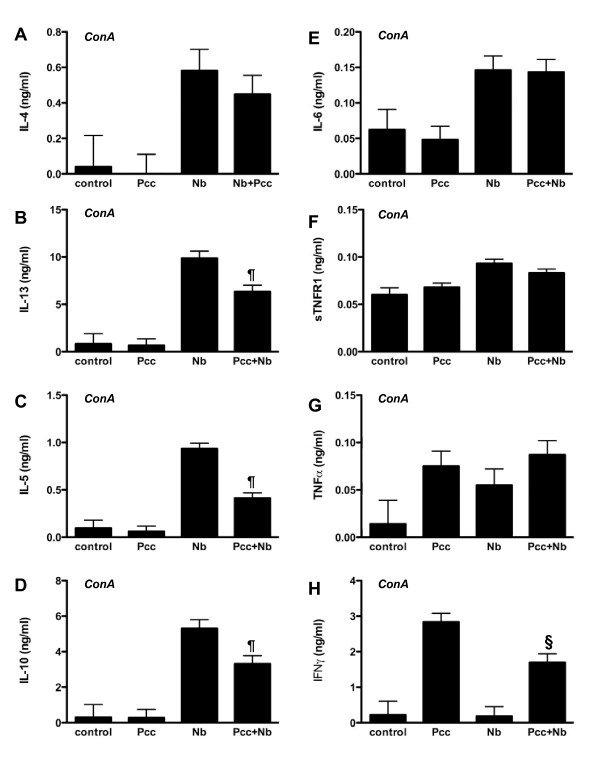
**ConA-induced cytokine release in TLN lymphocyte recall assays**. Cytokine concentrations in supernatants of ConA-stimulated TLN cells harvested at day 7 pi were measured with cytometric bead arrays. Groups did not differ in cytokine production at 3 or 5 days pi, and no antigen-specific responses were detected until 20 days pi. Data on day 7 pi IL-4 (A), IL-13 (B), IL-5 (C), IL-10 (D), IL-6 (E), sTNFR1 (F), TNF-α (G), and IFN-γ (H) production are shown. In these data, statistically significant down-regulatory effects of co-infection (*Nb+Pcc*) were found for IL-13 (*P *= 0.011), IL-5 (*P *= 0.0001), IL-10 (*P *= 0.007), and IFN-γ (*P *= 0.006), compared to relevant single-infection groups. Bars indicate mean ± SEM from 3 combined experiments, each with 4-9 mice per group. The symbol ¶ indicates significance of the difference between co-infected and *Nb *mice, while § indicates significance of the difference between co-infected and *Pcc *mice (*P *values included above and in Results text).

At 7 days pi, *Nb *infection in singly as well as co-infected mice was associated with significantly elevated ConA-induced production of IL-4, IL-13, IL-5, and IL-10 compared to uninfected and *Pcc*-infected mice (Fig. 8A, B, C, D; all *P *for *Nb *main effect < 0.0001). Of interest, IL-6 and sTNFR1 were also associated with *Nb *but not *Pcc *infection (Fig. 8E, F; both *P *for *Nb *main effect <0.0001). TNF-α production did not vary among infection groups (Fig. 8G), but *Pcc *infection was associated with significantly elevated IFN-γ production compared to uninfected and *Nb*-infected mice (Fig. 8H; *P *for *Pcc *main effect <0.0001). To our knowledge, TLN polarization by systemic malaria infection has not been previously reported. The Th1 bias of the observed response to *Pcc *was unsurprising.

Co-infected mice differed from singly-infected mice in ConA-induced production of some of these cytokines. For example, IL-13, IL-5, and IL-10 production were significantly reduced in co-infected compared to *Nb*-infected animals (Fig. 8B, C, D; t_61 _= 7.3, 18.8, 8.1 and *P = *0.011, 0.0001, 0.007, respectively), while IFN-γ production was reduced in co-infected compared to *Pcc*-infected animals (Fig. 8H; t_61 _= 8.4 and *P *= 0.006). These results provide evidence of cross-regulation between Th1 and Th2 immune responses in the local lymph node during the first week of co-infection. This finding is consistent with a wide range of studies of murine co-infection (reviewed in [30,46]).

**Figure 9:**
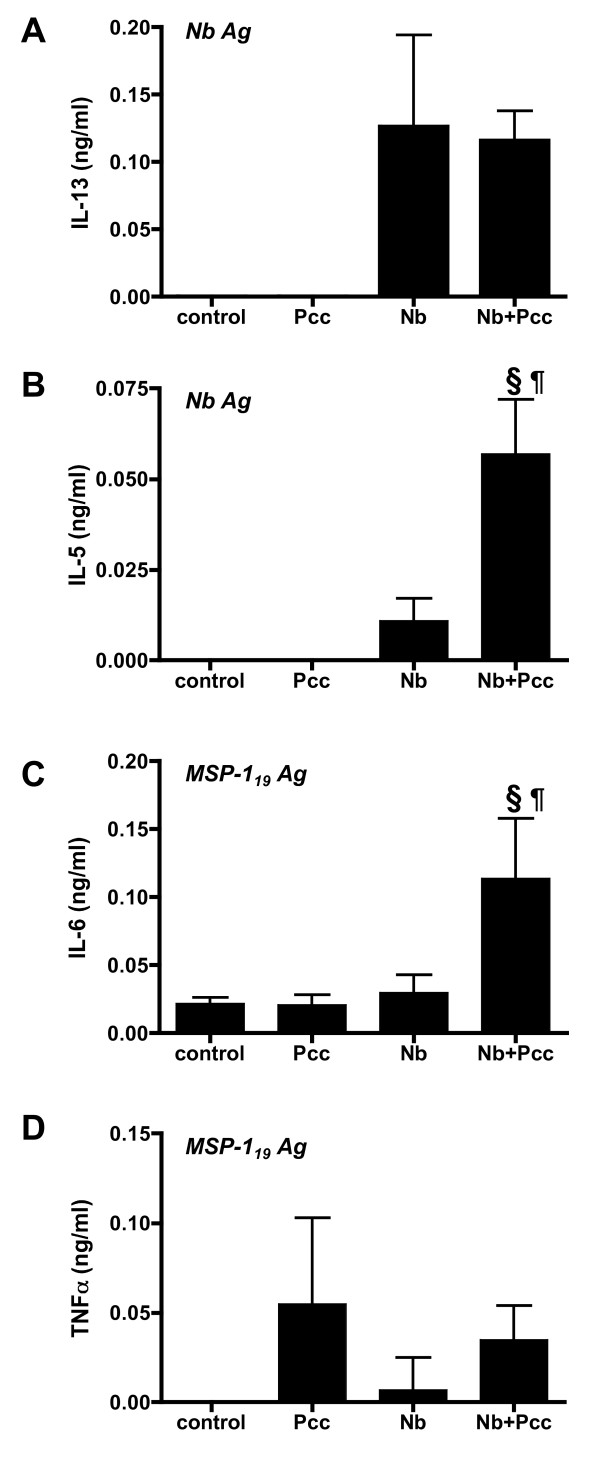
**Antigen-specific responses in TLN lymphocyte recall assays**. Cytokine concentrations in supernatants of TLN cells that were harvested at day 20 pi and then stimulated with antigen were measured with cytometric bead arrays. *Nb* Ag, a crude *Nb *adult lysate, and recombinant *Pcc *clone AS merozoite surface protein, MSP-1_19_, were used as antigens *in vitro*. Although all cytokines shown in Figure 8 were measured, only IL-13 in response to *Nb* Ag (A), IL-5 in response to *Nb* Ag (B), IL-6 in response to MSP-1_19 _(C), and TNF-α in response to MSP-1_19 _(D) were above levels of spontaneous secretion. These figures thus depict antigen-specific cytokine (in ng/ml above background). Statistically significant differences among groups included increased NbAg-specific IL-13 in all *Nb *mice (*P *< 0.0001), increased *Nb* Ag-specific IL-5 in co-infected (*Nb+Pcc*) mice compared to both *Nb *(*P *= 0.0025) and *Pcc *mice (*P *< 0.0001), and increased MSP-1_19_-specific IL-6 in *Nb+Pcc *compared to both *Nb *(*P *= 0.0163) and *Pcc *mice (*P *= 0.0112). Bars indicate mean ± SEM for 4-8 mice per group. The symbol ¶ indicates significance of the difference between co-infected and *Nb *mice, while § indicates significance of the difference between co-infected and *Pcc *mice (*P *values included above and in Results text).

By 20 days pi, the overall strength of TLN cytokine responses had waned, but antigen-specific responses were detectable. Correcting for background (spontaneous) secretion of cytokines in wells with media alone, antigen-specific responses to crude helminth (*Nb *Ag) and recombinant malaria (MSP-1_19 _Ag) antigens were each observed for a subset of the cytokines measured (Fig. 9). For example, *Nb *infection was associated with strong *Nb *Ag-specific IL-13 responses (Fig. 9A: *P *< 0.0001), regardless of co-infection. Co-infection, however, had a significant boosting effect on *Nb *Ag-specific IL-5 (Fig. 9B: *P *= 0.0025 for the comparison with *Nb *mice; *P *< 0.0001 for the comparison with *Pcc *mice). Co-infection was also associated with significantly elevated MSP-1_19_Ag-specific IL-6 (Fig. 9C: *P *= 0.0163 for the comparison with *Nb *mice; *P *= 0.0112 for the comparison with *Pcc *mice). TNF-α production, though greater than background, did not differ significantly among groups (Fig. 9D).

## Discussion

Our primary aim in this study was to address the interplay of two acute infections that place conflicting demands on the host immune response, particularly in the lung. We wanted to focus on Type 1-Type 2 cross-regulation rather than any effects of regulatory T cells, so we opted for a model of acute rather than chronic helminthiasis. In addition, although anatomical compartmentalization does not preclude immunological interaction - for example, gut-restricted helminths can induce a strong systemic Th2 bias [47] - compartmentalization can buffer the effects of co-infection [48]. We thus chose murine infection models that would pose an immunological conflict in anatomical space as well as time post-infection (pi). The dynamics we were investigating may have real life corollaries, because nematode migration occurs in the lungs of over a third of the world's human population [1,2], many of whom are co-infected with malaria [31,32]. However, because both of our murine models (*Pcc *and *Nb*) produce self-resolving infections, the effects of co-infection on anaemia and on pulmonary immunology reported here cannot be firmly associated with chronic disease outcomes until longer term co-infection studies are performed.

We quantified the health of mice in our experiments using two measures that have proven informative during *Pcc *infection [49] and *Pcc*-nematode co-infection [50]: body mass and RBC density. To our knowledge, this is the first report demonstrating that murine *Nb *infection has a negative impact on both parameters, although reduced weight gain in young *Nb*-infected mice has previously been reported [40]. *Nb *caused a statistically significant, transient ~3% loss of body mass from approximately 2-4 days pi, and in other experiments using a higher dose (500 L3s), mice lost closer to 10% of their starting body mass (unpublished data). Migration of *Nb *larvae through the lungs has previously been shown to cause two spells of inappetance and thus weight loss in rats, one associated with migration of larvae and the other with establishment of adults in the gut [41]. We detected only one period of loss of body mass; mice may be spared the second spell given the brief survival of adult *Nb *in mice, particularly for parasite strains, such as ours, that are not mouse-adapted. We also observed a transient loss of RBC density in *Nb*-infected mice. It was rather surprising that this effect - most likely caused by haemorrhaging of the lung following larval migration - was detectable at the systemic level. This suggests that the capillary damage and ingestion of RBCs by alveolar macrophages following lung migration of *Nb *[11,16] are associated with considerable blood loss.

A diverse range of outcomes is possible when helminths and malaria co-infect a host. Co-infected mice in our study experienced two periods of RBC loss in quick succession - first *Nb*-induced and then *Pcc*-induced. However, they had slightly higher RBC densities than *Pcc*-infected mice did, at the time of most severe malarial disease. This was associated with a small reduction in malaria parasitaemia in the blood. These results contrast with several studies of helminth-malaria co-infection in mice, in which malaria parasitemia was increased [51-54], and/or malarial symptoms exacerbated, in at least some groups of co-infected mice [50-55]. For example, in contrast to the lethal inflammatory liver disease recently described in mice simultaneously co-infected with *Heligmosomoides polygyrus *and *Pcc *[55], we observed subtle amelioration of malarial disease and no deaths. This disparity in the severity of co-infection could be due to the fact that we worked with a different mouse strain (BALB/c versus Helmby's C57BL/6) as well as a different helminth species that migrates differently through the host body. However, we detected an elevation in MSP-1_19_-specific IL-6 due to co-infection, so it is possible that the emergent IL-17/IL-23 axis described by Helmby [55] may likewise be involved in our co-infection system, though not in organs that negatively impact short-term survival. Indeed, the mechanisms underlying the slightly protective effect of *Nb *observed here are not yet clear. We are investigating possible immunological causes of this protection, including innate mechanisms such as IFN-γ^+ ^NK cells [56] and adaptive mechanisms such as cytophilic antibody isotypes [33] that could promote malaria clearance; either might be altered by acute *Nb *co-infection. However, it is also possible that lower parasitaemia might be the consequence of the RBC density changes induced by *Nb*, as previous co-infection studies have shown that helminths can limit RBC availability to malarial parasites and thereby cap their replication (e.g., [57]). Control of microparasites by Th1 immunity and by RBC limitation are not mutually-exclusive possibilities [58] and both might be operating in our model system. Finally, it is possible that the sequestration habits of *Pcc *parasites [59] are altered by *Nb*. These mechanisms remain to be investigated.

Our results largely resemble those reported for other *Nb*-microparasite pairings. For example, during co-infection of mice with *Nb *and either *Toxoplasma gondii *[60] or *Chlamydophila abortus *[61], significantly reduced Th2 responses (compared to mice with *Nb *infection) have been observed, independent of the interval between infections [60,61]. These data suggest that *Pcc *is not the only microparasite that might reduce Th2 responses to *Nb *infection. *Mycobacterium bovis *BCG co-infection, however, does not significantly impact *Nb*-induced IL-4 in the mesenteric lymph nodes [62]; it would be of interest to know whether those lung-dwelling microparasites might have had similar effects to *Pcc *on Th2 responses, had they been measured in the TLN. Reported effects of *Nb *on the course of microparasite infections are likewise mixed: densities of *T. gondii *[60] and *M. bovis *BCG [62] are unaffected by the presence of the nematode, while *C. abortus *density increases dramatically [61]. Interestingly, the concurrent presence of influenza virus with migrating *Nb *larvae in the lung exacerbates the severity of lung disease compared to mice with influenza alone [40]. A two-week delay between *Nb *infection and influenza infection, or replacement of *Nb *with *H. polygyrus*, eliminates the added pathology, suggesting that the simultaneous presence of larvae and virus in the lungs is required [40]. Such may also be the case for *Pcc-Nb *co-infection. The *Nb*-influenza study did not include immunological measurements, so the role of the immune system in generating the observed pattern is not known. Indeed, this comparison illustrates that many details of anatomical location and parasite life cycles, as well as immunological interactions, must be taken into account to explain the diverse outcomes of helminth-microparasite co-infections [30,46].

Our most novel finding is that malaria infection has the capacity to modulate the host's pulmonary Type 2 response to nematode migration. However, the long-term impact of the altered Type 2 response is not possible to predict, because the function of Type 2 immunity in this setting is not yet fully understood. There are at least three potential outcomes of a helminth-induced Type 2 response in the lung. First, it may contribute to protection against incoming larvae [6]. Second, Type 2 responses are likely to be involved in repairing the damage that is inflicted by migrating parasites. Third, as recent studies have shown [5,16,18], lung migration and the associated Th2 responses have the potential to cause long-term lung pathology. Appropriate repair versus lung malfunction are likely to be flip sides of the same coin. Indeed, although ChaFFs and Arginase-1 are implicated in tissue repair, they are also associated with fibrosis, an overzealous repair process [19,24,43,63-65] (see also review by Wynn [66]). Predicting the effect of *Pcc *co-infection on long term *Nb*-induced lung disease is further complicated by recent data that suggest both Arginase-1 and RELMα can negatively regulate Th2-mediated pathology [27-29]. By this logic, inhibition of these molecules by malaria co-infection may ultimately exacerbate Th2-mediated lung damage.

However, our data suggest that the effect of malaria on ChaFF expression is not direct but rather via reduced Th2 cytokines. The effect of *Pcc *on *Nb*-induced ChaFFs was not apparent until 7 days pi, when the extent of the increase in ChaFF expression was inhibited by co-infection. This was correlated with differences in cytokine production in lymphocyte recall assays, suggesting that changes in ChaFF expression were driven by changes in the T lymphocyte populations after the onset of the adaptive Th2 immune response (around 5 days pi, as shown in *Nb-*infected IL-4 reporter mice [9]). A role for adaptive immunity is further supported by work showing that SCID mice are not able to sustain AAMφ responses in the lung following *Nb *infection [11], and a demonstrated requirement for T cells to sustain the AAMφ response in a mouse peritoneal infection model [24]. Remarkably, in SCID mice, in the absence of T cells and AAMφ, the *Nb*-induced cellular infiltrate does not resolve [11]. The capacity of malaria to inhibit the transition to a full Th2 response by 7 days pi may likewise be detrimental to full resolution of the inflammatory response, a step necessary for appropriate tissue repair [67,68]. By day 20 pi, however, the residual Th2 responses in co-infected mice were as high as, or even higher than, in *Nb*-only mice. In support of this, day 20 antigen-specific IL-5 responses were particularly high in co-infected animals. Thus *Pcc *infection may protect against airway hyper-responsiveness through a reduction in peak Th2 activation, or else exacerbate it due to sustained Th2 activity. Transient passage of *Nb *larvae through the lung inflicts lasting damage [16,18]. Whether transient impairment of pulmonary Th2 responses by malaria co-infection also has lasting effects needs to be investigated experimentally.

A perhaps surprising finding in our study was the apparent absence of classical macrophage activation in the lung despite the clear presence of malaria parasites: we did not detect iNOS, IL-12p40 nor elevated TNF-α mRNA in lung tissue of *Pcc*-only or co-infected mice at any time point. One could argue that malaria parasites stay in the lung microvasculature and do not cross into tissue. However, this is unlikely to be the case, given the extensive lung damage due to *Nb *in co-infected mice, as well as evidence that malaria merozoites can be found dispersed in the lung [34]. Furthermore, IFN-γ mRNA was detectable in the lung of all *Pcc *mice regardless of co-infection, suggesting that lymphocytes were activated, perhaps by innate activation of NK or γδ T cells. The most likely explanation for the failure to detect classical macrophage activation may be that lung macrophages, which are exposed daily to inhaled microbes, have a remarkably high threshold for activation even in the presence of IFN-γ and microbial stimuli [69].

As with any laboratory model, it is important to acknowledge the potential disconnection between natural co-infections and the experimental systems and designs used here, including the relative timing of the two infections, doses at which they were administered, and the fact that we have only studied primary and self-resolving (rather than secondary and/or chronic) infections. Permutation of any of these parameters is likely to quantitatively, if not qualitatively, alter outcomes. For example, repair processes might readily keep pace with lung damage when the rate of exposure to nematode larvae is low, unlike in most experimental models. We used a relatively low dose of L3 larvae (200 per mouse while others use ~500 [16,18] or as many as 750-1000 [60-62]) but still exceeded natural exposure levels. Furthermore, larval helminths and malaria parasites are unlikely to arrive in the lung within a few days of each other in nature, and it may be that pre-existing malaria would have had a different effect on pulmonary Type 2 responses to *Nb *migration, particularly if malaria parasites do not remain long in the lung. Indeed, the most likely natural exposure scenario may be chronic malaria infection into which helminth larvae are "trickled" [32], but experimental studies that mimic this scenario have yet to be carried out. Nonetheless, lung dysfunction is seen as a consequence of helminth migration [4,5] and both acute and persistent malaria infection [38] in people, so high-dose experimental *Nb *studies in which long-term lung pathology can be observed [16,18], combined with simultaneous malaria exposure, may provide useful models for disease states in people.

## Conclusion

With the experiments reported here, we have established an acute laboratory model of helminth-malaria co-infection that will be suitable for future work exploring the details of how Type 1 inducing co-infections affect long-term, Type 2-mediated repair of the damage caused by migrating nematodes. Recently developed models of malaria-induced lung damage (e.g., [37]) might be analysed in animals co-infected with *Nb*. Corroborative studies in human populations may also be feasible. Like migratory helminthiases [3], severe falciparum malaria is associated with detectable lung injury, as measured by spirometry and clinical symptoms [38]. A study like Brooker *et al*'s analysis of whether the anaemia of hookworm and malaria are additive during co-infection [32] that used spirometry to assess the pulmonary health of malaria-infected, *A. lumbricoides- *or hookworm-infected, and co-infected people could assess whether co-infection exacerbates damage. Given the huge number of people with such co-infections, it is possible that clinical studies of malaria lung injury may gain insight from considering the presence, however transient, of helminths in the lung.

## Methods

### Mice, parasites, experimental design, and monitoring

Specific pathogen free, 8-10 week old female BALB/c mice (Harlan, UK) were maintained in individually ventilated cages on diet 41b *ad lib*. *Nippostrongylus brasiliensis *(*Nb*) was maintained by serial passage through Sprague-Dawley rats, as described previously [70]. Cryopreserved *Plasmodium chabaudi chabaudi *(*Pcc*) parasites of clone AS were passaged through two generations of donor BALB/c mice and inoculated into experimental mice as described previously [50]. The four co-infection experiments used a factorial design, with uninfected controls, *Pcc*-infected, *Nb*-infected, and co-infected mice. On day 0, 200 *Nb *L3 larvae and/or 10^5 ^*Pcc*-infected RBCs were injected subcutaneously and intraperitoneally, respectively. PBS and naïve mouse RBCs served as sham injections for *Nb *and *Pcc*, respectively; uninfected control animals received both sham injections. RBC density, body mass, and malaria parasites were then monitored daily, as described previously [50]. Briefly, RBC densities were measured by flow cytometry (Beckman Coulter), body mass was recorded to the nearest 0.1 g, and the proportion of RBCs parasitized was counted in Giemsa-stained thin blood films (at 1000× magnification). Mice were then culled 3, 5, 7, or 20 days pi (4-9 mice per infection type per timepoint). *Nb *parasite burden was assessed in the gastrointestinal tract of culled mice. Intestines were placed in PBS, slit lengthwise and the contents rinsed into muslin-lined funnels set over tubes containing PBS warmed to 37°C. Nematodes were left to filter through for >2 hours and counted via microscopy (at 40×). To elucidate the dynamics of *Nb*-induced alternative activation in the lung, a separate experiment was conducted for *Nb *only, with mice culled 3, 5, 7, 15, 20 or 26 days pi (4 *Nb*-infected mice per time point). All experiments were carried out in accordance with the animals (Scientific Procedures) Act 1986, and were approved by the UK Home Office inspectorate and institutional review committee.

### Lung lavage and tissue sampling

Following terminal anaesthesia, tracheas were cannulated and lungs lavaged with 1 mL PBS. Cannulae were prepared from fine bore polythene tubing (Portex) and a 23 G needle. Following lavage, the left lung lobe was tied off, cut at the bronchus, and placed in RNAlater (Ambion) for mRNA extraction, while the right lobe was perfused in 4% formaldehyde and embedded in paraffin for immunohistochemistry. BAL cell concentrations were determined using a Scharf Instruments Casy Counter. BALF was centrifuged at 1,200 g for 5 mins and stored at -20°C for protein analysis by Western blot.

### Immunohistochemistry

Expression of RELMα and Ym1 in lung sections was assessed by indirect immunoperoxidase techniques. Briefly, the paraffin embedded tissue sections were deparaffinised and rehydrated. After high temperature antigen unmasking (Vector Laboratories, UK), endogenous peroxidase was quenched with aqueous 2% H_2_O_2 _(Sigma Aldrich, UK) for 15 minutes. Slides were then incubated 2 h with primary antibodies: rabbit anti-RELMα (0.25 μg/mL; Peprotech) or rabbit anti-Ym1 (1/100; StemCell Technologies) in antibody diluent (Dako Cytomation, Denmark) at RT, followed by the secondary antibody (goat anti-rabbit biotin, 1 mg/mL, Dako Cytomation, Denmark). Peroxidase-labelled ABC reagent and DAB substrate (Vector Laboratories, UK) were used for signal visualisation. Finally, the sections were counterstained with haematoxylin. RELMα and Ym1 staining intensities were scored by two researchers, blinded to experimental groupings, using a modification of a previously-published lung inflammation scoring system [71]. For each mouse, staining was assessed at 200× magnification for 10 fields. Each field included correctly inflated lung tissue and a complete transection of at least one bronchiole, blood vessel and alveolar airway. Cytoplasmic staining strength was scored in bronchial epithelial cells, infiltrating cells and alveolar macrophages on a scale of 1-4 (1 = no staining, 2 = weak, 3 = moderate, and 4 = strong staining, using a reference section of the same positive control sample (lung of an *Nb*-infected mouse at 7 days pi). The percentage of positive cells in each of these compartments was also scored on a scale of 1-4 (1 = none, 2<30%, 3 = 30-60%, 4>60% positive cells). Average cytoplasmic and cell positivity scores across the 10 fields were calculated. Finally, the overall staining score for each mouse was calculated by multiplying the average stain strength by average % positive cells. Control sections incubated with antibody diluent followed by secondary antibody only, or with normal rabbit serum alone, did not show any staining. Mouse lung pathology experts confirmed that stained cell types were correctly identified. Photomicrographs of representative sections were captured on a Zeiss Axioskop microscope with QCapture Pro Software.

### RNA isolation and real-time RT-PCR

RNA isolation from lung tissue was carried out using TRIzol (Invitrogen). After DNase treatment (10 U/mL DNase1, Ambion), cDNA was synthesised using Moloney murine leukaemia virus reverse transcriptase (Stratagene). For quantification of Ym1, RELMα, Arginase 1, iNOS, IL-12p40, TNF-α, IFN-γ and IL-13 mRNA, real-time RT-PCR was performed using a LightCycler (Roche Diagnostics) and primers reported previously [63]. For each gene, five serial 1:4 dilutions of cDNA of a positive control sample (for RELMα, Ym1 and Arg1, peritoneal macrophages of a mouse implanted with *Brugia malayi *adult parasites for 3 weeks [72]; for IL-13, spleen of an MHV-68 infected IFNγR-KO mouse [63]; or for iNOS and other Type 1 markers, peritoneal macrophages of a thioglycolate-injected mouse [10]) were used in each reaction. Amplification was quantified and normalised using β-actin as a housekeeping gene. PCR reactions were carried out in 10 μl buffer containing 1 μl cDNA, 4 mM MgCl_2_, 0.3 μM of each primer and the LightCycler-DNA SYBR Green I mix, under the following conditions: 30 s denaturation at 95°C, 5 s annealing of primers at 55°C or 63°C (Ym1), and 12 s elongation at 72°C, for 50 cycles. SYBR Green fluorescence was monitored after each cycle at 86°C (85°C for Ym1).

### Real-time PCR to detect malaria in lung tissue

Real-time PCR for *Pcc *genomic DNA was carried out on DNA extracted with phenol/chloroform from homogenized lung tissue (stored in Trizol, Invitrogen; 75 mg tissue/mL). PCR was performed on an ABI Prism 7000 (Applied Biosystems), with primers for merozoite surface protein (MSP)-1 of clone AS, as described previously [73].

### Western blot for Ym1 and RELMα

20 μl BALF was mixed with sample buffer supplemented with denaturing buffer (NuPage, Invitrogen), heat denatured and resolved by SDS-PAGE using 4-12% gradient Bis-Tris gels (NuPage, Invitrogen) followed by transfer onto nitrocellulose membrane (Bio-Rad). Transfer and loading intensity were assessed with Ponceau Red staining (Sigma). After blocking with 0.05% Tween 20 in Starting Block (Pierce), membranes were incubated overnight at 4°C with polyclonal rabbit anti-Ym1 [10] (0.12 ng/mL) or rabbit anti-RELMα (0.2 μg/mL; Peprotech). After incubation with HRP-conjugated goat anti-rabbit IgG (heavy plus light chains; Bio-Rad; 1/2000), signal was detected with chemiluminescence (ECL kit, Amersham Pharmacia Biotech) and exposure to Hyper ECL film (Amersham). Control blots were incubated with secondary antibody only. Band intensity was determined with the FluorChem SP imager system and software (Alpha Innotech, USA) and expressed as percentage relative to a positive control (pooled lung lavages of three *Nb*-infected mice) on each blot.

### Measurement of cytokine and cytokine receptor responses in local lymph nodes

Thoracic lymph node (TLN) cells were cultured at 5 × 10^5 ^cells per well, with 1 μg/mL Concanavalin A (ConA), 10 μg/mL adult *Nb *parasite extract, 1 μg/mL of recombinant *Pcc *Merozoite Surface Protein MSP-1_19_, or medium alone at 37°C. After 72 h, supernatants were harvested. Concentrations of IL-4, IFN-γ, TNF-α, IL-5, IL-6, IL-10 and IL-13 were then measured using Cytometric Bead Array Flex Sets (BD Biosciences), with slight modifications from manufacturer's instructions: 50 μl samples/standards were incubated with capture beads (0.5 μl per sample per cytokine, plus diluent to 25 μl) in darkness with shaking for 1 h at RT. Plates were washed, spun at 200 g for 5 min, and then incubated with 25 μl of PE-conjugated anti-cytokine antibodies in darkness for 1 h. After washing and resuspension of beads, data were acquired on a FACSArray with FCAP software (BD Biosciences). Soluble TNF Receptor-1 (sTNFR1) concentrations were determined by sandwich ELISA, using 2 μg/mL capture antibody (clone MAB425), 200 ng/mL biotinylated detection antibody (BAF425), and recombinant mouse sTNFR1 as standard (425-R1; all from R&D Systems). Plates were blocked with 5% BSA in TBS for 2 h at RT and washed with TBS/0.05% Tween (TBST) before 50 μl samples were incubated overnight at 4°C. Plates were washed, incubated with detection reagent for 2 h at RT, washed again, and then incubated with streptavadin-HRP (Sigma Aldrich) at RT for 20 min. Plates were washed again and developed with TMB SureBlue substrate system (KPL 52-00-03). The reaction was stopped after 30 min with 1 M HCl and read on a spectrophotometer at 450 nm.

### Statistical analysis

Most data were analysed with SAS System 9.1 mixed-model analyses of variance (ANOVA) or covariance (ANCOVA) [74] (see exceptions below). To meet the homogeneity-of-variance assumption of such analyses, data were logarithmically transformed. To account for slight differences among experiments in, for example, the magnitude of ConA-stimulated cytokine production, *experiment *was included as a random factor in all models. All significant effects of *infection *reported below have therefore remained significant after controlling for effects of *experiment*, if any. To account for differences among mice in starting body mass or RBC density, day 0 values were included as covariates. Pathology analyses focused on animals that experienced the full 20-day course of infection, and minimum body weight and RBC density were analysed in two time frames: the first week pi, and the entire experiment. In accord with the factorial design of the experiments, *Pcc *and *Nb *infection were fit as fixed factors. Wherever the interaction term was significant (indicating a potential effect of co-infection), post-hoc t-tests adjusted for multiple comparisons were run. A Tukey-Kramer-corrected *P *< 0.05 was used as the cut-off for significance. For relevant subsets of mice, an *Nb *fixed factor was used to test whether co-infection altered *Pcc *parasitaemia, while a *Pcc *fixed factor tested whether co-infection altered expression of ChaFFs.

Following logarithmic transformation to meet the assumptions of parametric statistical analysis, the ChaFF time courses, IHC scores, and lung *Pcc *data were analysed with unpaired *t*-tests in Prism 4 (Graph Pad Software, Berkeley, USA), with a two-tailed *P *< 0.05 designated as significant. Only two groups were compared per dataset, as specified in the Results section.

## Abbreviations

AAMφ: (alternatively activated macrophages); BALF: (bronchioalveolar lavage fluid); ChaFFs: (chitinase and Fizz/resistin family members); IHC: (immunohistochemistry); IL: (interleukin); *Nb*: (*Nippostrongylus brasiliensis*); *Pcc*: (*Plasmodium chabaudi chabaudi*); pi: (post-infection); RBC: (Red Blood Cell); RELMα: (resistin-like molecule α); Th: (T helper); TLN: (thoracic lymph node).

## Authors' contributions

MAH conducted RT-PCR analysis of ChaFFs and PCR analysis of *Pcc *genomes, ran Western blots, statistically analysed some of the data, assisted with IHC scoring, and helped to draft the manuscript. KJM led the lung sampling, conducted cytokine RT-PCR reactions, assisted with Western blots, did IHC staining and scoring, and helped to draft the manuscript. Additionally, KJM collected all data for the *Nb *timecourse experiment. KJF-C, aided by ALG, set up all co-infection experiments and collected parasitemia, body mass and anaemia data. SM, aided by KJF-C, cultured lymph node cells and measured cytokines and cytokine receptors in the supernatants. JEA and ALG conceived of and designed the study, and drafted the manuscript. ALG performed most of the statistical analysis. All authors contributed to scientific discussions of the data, read and approved the final manuscript.
